# Targeted p53 on Small-Molecules-Induced Ferroptosis in Cancers

**DOI:** 10.3389/fonc.2018.00507

**Published:** 2018-11-02

**Authors:** Weifen Zhang, Chengcheng Gai, Dejun Ding, Fang Wang, Wentong Li

**Affiliations:** ^1^Department of Pharmacology, Weifang Medical University, Weifang, China; ^2^Department of Pathology, Weifang Medical University, Weifang, China; ^3^School of Clinical Medicine, Weifang Medical University, Weifang, China

**Keywords:** p53, ferroptosis, reactive oxygen species, tumor suppression, metabolic gene

## Abstract

Ferroptosis is a type of programmed cell death characterized by the accumulation of lipid reactive oxygen species (L-ROS) driven by the oxidative degeneration of lipids in an iron-dependent manner. The mechanism by which lipid oxidative degradation drives ROS-ferroptosis involves metabolic dysfunctions that result in impaired intracellular metabolic processes and ROS production. Recent studies have found that p53 acts as a positive regulator of ferroptosis by promoting ROS production. p53 directly regulates the metabolic versatility of cells by favoring mitochondrial respiration, leading to ROS-mediated ferroptosis. In mild stress, p53 protects cell survival via eliminating ROS; additionally, in human colorectal cancer, p53 antagonizes ferroptosis by formation of the DPP4–p53 complex. In short, the mechanisms of p53-mediated ROS production underlying cellular response are poorly understood. In the context of recent research results, the indistinct roles of p53 on ROS-mediated ferroptosis are scrutinized to understand the mechanism underlying p53-mediated tumor suppression.

## Introduction

Ferroptosis, a new form of cell death, was first described in a high-throughput screening of molecules for selectively inducing cell death in RAS mutant isoform cancer cells (1). As a novel subtype of programmed cell death, ferroptosis is primarily characterized by increased mitochondrial membrane density and volume shrinkage with distinct morphological, biochemical, and genetic differences from other types of cell death, including apoptosis, necrosis, necroptosis, and autophagy; for instance, the typical characteristics of apoptosis, including activated caspases, chromatin condensation, and DNA fragmentation, are absent in ferroptosis (1, 2), the distinctive morphological feature of erastin-treated cells involved smaller mitochondria with increased membrane density (3). In addition, loss of the plasma membrane integrity of necrotic morphological features and formation of double membrane-layered autophagic vacuoles during autophagy are not observed in ferroptosis.

Small molecules belonging to class I and class II ferroptosis-inducing agents trigger ferroptosis via inhibiting cystine-glutamate exchange transporter ($system\;X_{c}^{-}$) and glutathione peroxidase 4 (GPX4), respectively (4). Class I ferroptosis inducers, such as erastin, sorafenib, sulfasalazine and the neurotransmitter glutamate, $system\;X_{c}^{-}$, class II ferroptosis inducers, such as RSL3, FIN56 (5), or altretamine (6) are shown to induce ferroptosis via inhibition of GPX4.

Recent studies have reported that p53 activation is essential for ferroptosis in certain cancers. Since the discovery of p53, its role on tumor suppression in tumorigenesis and cancer therapy has attracted considerable attention. Loss of p53 is a vital event in the tumorigenesis of many human cancers (7, 8). In general, the tumor suppression activity of p53 in response to cellular stress relies on its capability to elicit cell-cycle arrest, apoptosis, and senescence. Nevertheless, recent efforts indicate that other unconventional activities of p53 are also crucial for tumor suppression (9, 10).

Novel roles of p53 on tumor suppression have come to light when a synthetic mutant of p53, incapable of transactivating the majority of known p53 target genes, displays antitumor activities in unstressed organisms and some cancer-prone mouse models (10, 11). A mutant p53 that loses acetylation at some definite residues of the DNA binding domain is disabled to evoke growth arrest, senescence, and apoptosis, thereby inhibiting spontaneous tumor development through sensitizing cells to ferroptosis (12, 13). Given that p53 is a main regulatory factor of critically important cellular biological processes, elucidating the mechanism by which p53 responds to stress may clarify the upstream signaling of ferroptosis. In the context of recent insights, the indistinct roles of p53 signaling in reactive oxygen species (ROS)-mediated ferroptosis via the transcriptional and non-transcriptional regulation of metabolic targets are scrutinized (Table 1).

**Table 1:** The mechanisms of transcriptional and post-translational regulation on metabolic genes involving in ferroptosis.

**Active style**	**Targets**	**Function**	**References**
Transcriptional regulation	GLUT1, GLUT4	Negatively regulates glycolysis by transcriptional repression	(14)
	TIGAR	Negatively regulates glycolysis by transactivation	(15–17)
	GLS2	Favoring aerobic glycolysis over oxidative phosphorylation and contributing to Warburg metabolism	(11, 18–20)
	SCO2	Coupling p53 to mitochondrial respiration provides a possible interpretation for the Warburg phenomenon	(13, 21)
	SLC7A11	Repression of SLC7A11 leads to destruction of cystine import, resulting in declined glutathione production and enhanced ROS-mediated ferroptosis	(9, 15)
	RRAD	Negatively regulates glycolysis	(17)
	SAT1	lipid peroxidation and ROS-induced ferroptosis	(22)
	p21	Slower depletion of intracellular glutathione and a reduced accumulation of toxic L-ROS	(23)
Post-translational regulation	G6PDH	Suppress glucose metabolism directly via binding and inhibiting with G6PDH	(24)
	DPP4	Dismantling of DPP4-p53 complex	(25)
	SOSC1	The regulation of SAT1 by p53 was SOCS1-dependent, stabilizating p53	(26)

## Activation of p53 sensitizes cells to ROS and triggers ferroptosis

Increased accumulation of lipid reactive oxygen species (L-ROS) in an iron-dependent manner is a fundamental characteristic of ferroptosis (14, 27). Metabolic dysfunctions contribute to ferroptosis by elevating the production of ROS independent of mitochondria (5). Thus, several investigations have been devoted to elucidate the regulatory roles of p53 on metabolic targets in ROS production for regulating ferroptosis.

**Figure 1:**
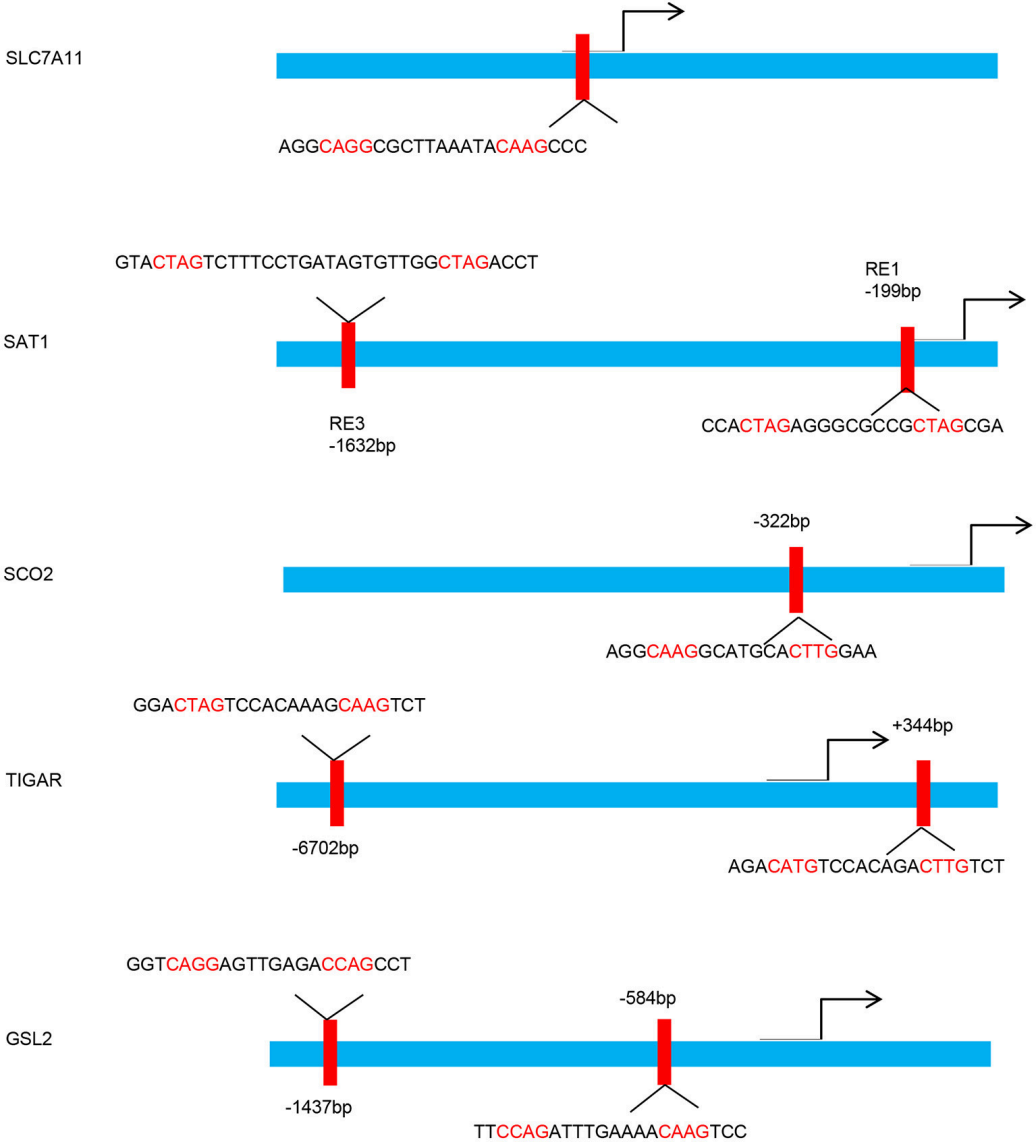
p53 binding sites within the upstream regulatory region of the target gene promoters. Schematic diagram indicates the p53 binding sites within the upstream regulatory region of the SLC7A11, SAT1, SCO2, TIGAR, and GSL2 promoters.

p53 participates in various cellular processes by acting as a DNA binding transcription factor that selectively modulates the expression of target genes. For example, wild-type p53 regulates the transactivation of cytochrome c oxidase 2 (SCO2), favoring mitochondrial respiration over glycolysis (28). In addition, p53 plays a negative regulatory role on glycolysis via transcriptionally modulating glucose transporter (GLUT)1, GLUT4 (24), TP53-induced glycolysis and apoptosis regulator (TIGAR), and glutaminase 2 (GLS2) (15, 29) (Figure 1). p53 could also suppress glucose metabolism directly by binding and inhibiting glucose-6-phosphate dehydrogenase (30). Clearly, p53 directly adjusts the metabolic polyfunctionality of cells by supporting mitochodial respiration, leading to ROS-mediated ferroptosis.

## Modulation of p53 on the expression of SLC7A11 to mediate ferroptosis

### p53 represses SLC7A11 expression

SLC7A11 (xCT) is a light-chain subunit of the membrane Na^+^-dependent $system\;X_{c}^{-}$, which is a disulfide-linked heterodimer composed of SLC7A11 and a heavy-chain subunit (SLC3A2) (31). Previous experiments showed the inconformity in the p53 activation and expression of SLC7A11, which could directly affect ferroptosis in mouse embryonic fibroblast (MEF) cells (32). $System\;X_{c}^{-}\;$transfers intracellular glutamate to the extracellular space and extracellular cystine into cells (33). Cystine is then converted into cysteine for synthesizing glutathione (GSH), which protects cells from oxidative stress. Inhibition of $system\;X_{c}^{-}$ reduces intracellular GSH, resulting in an iron-dependent ferroptosis mediated by the accumulation of L-ROS (23).

**Figure 2:**
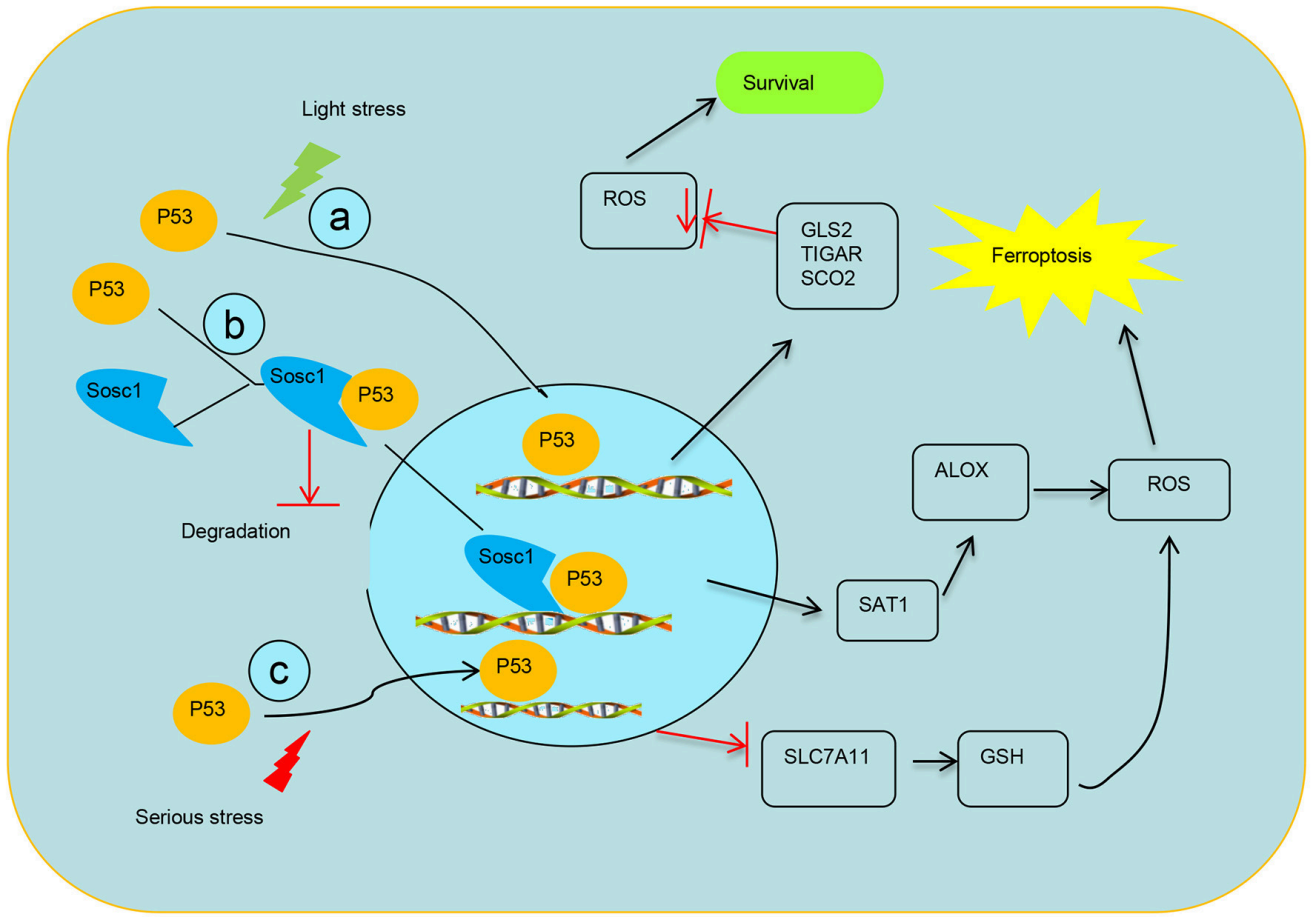
Schematic diagram of transcriptional regulation of p53 on targets. **(a)** p53 transcriptionally represses the expression TIGAR, GLS2, and SCO2 to mediate ferroptosis. **(b)** SOCS1 is required for p53 modulating some target genes and SOCS1–p53 complex preserves a pool of preactive p53 via preventing p53 degradation. **(c)** Modulation of p53 on the expression of SLC7A11 system ${\text{X}}_{\text{c}}^{-}$ activity to mediate ferroptosis.

Activation of p53 by nutlin-3 markedly decreases SLC7A11 expression in HT-1080 cells with basal $system\;X_{c}^{-}$ activity (34). Knockdown of p53 completely abrogates the inhibition of SLC7A11 (35), and $system\;X_{c}^{-}$ function and SLC7A11 expression in p53^KO^ cells are insensitive to nutlin-3 (36). Furthermore, microarray analysis confirmed that SLC7A11 is a novel target gene of p53 in a tetracycline-controlled p53-inducible cell line (13). A previous study identified a p53-binding sequence at the 5′ flanking region of the SLC7A11 gene and subsequently confirmed the formation of a p53–DNA complex at the promoter region (13). The transcriptional repression of p53 on SLC7A11 leads to the destruction of cystine import, resulting in declined glutathione production and enhanced ROS-mediated ferroptosis (Figure 2).

### p53-dependent repression of SLC7A11 is independent of p53 mutation

The molecular cascade whereby p53 restrains cystine transfer by suppressing SLC7A11 expression to induce ferroptosis may be conducive to the oncosuppressive roles of p53 (13). Although an acetylation-absent p53^3KR^ (K117/161/162R) variant at certain lysine residues cannot transcriptionally activate gene expression involved in pro-apoptotic and cell cycle arrest, knock-in mice expressing p53^3KR^ are not tumor prone and exhibit similar overall survival with the wild-type mice (12). Similarly, studies on p53^25, 26^, a transactivation-compromised mutant variant of p53, displayed intact tumor suppression of p53^3KR^ in the absence of the most downstream genes of p53 (10). Reduced levels of SLC7A11 expression caused by the p53^3KR^ variant in xenograft tumor models lead to an apparent depression of tumor growth (13). This finding indicates that the intact p53-SLC7A11 axis, reserved in the p53^3KR^ variant, promotes the inhibition of tumorigenesis independent of the conventional tumor suppression mechanisms of p53. Thus, ferroptosis can ensue from the transcriptional repression of SLC7A11 in a p53-dependent mechanism in response to stress, irrespective of p53 mutational status (37).

However, whether cell ferroptosis upon ROS-induced by p53^3KR^ in human cancer cells is similar to that of wild-type p53 remains unclear. In addition, whether cyclophilin D could be a downstream responder of p53 activation has yet to be clarified (38).

### Acetylation is crucial for p53-mediated ferroptosis

p53 activity is controlled by a complex fine-tuning network that includes protein stability, recruitment of co-inhibitor or activator, and various post-translational modifications, such as acetylation, ubiquitination, phosphorylation, and methylation (25, 39). In particular, acetylation of p53 serves a vital role in regulating downstream targets in a promoter-specific activation during stress responses. Acetylation of p53 at K120 by Tip60/MOF is crucial for p53-induced apoptosis (40). Nevertheless, p53-mediated cell cycle arrest is involved in the combinative acetylation of K120 by Tip60/MOF and K164 by CBP/p300 (41). The p53^3KR^ mouse expressing acetylation-deficient p53, similar to the K120/164R mutations in human, displays intact p53-dependent metabolic regulation but lacks p53 functions in pro-apoptosis activity and growth arrest (12).

A recent study has found that p53 is acetylated at lysine residue K98 by acetyltransferase CBP. Acetylation of p53 at K98 lysine residue in mouse does not interfere with the steady-state, DNA-binding abilities and transcriptional activity of p53. However, combinatorial absence of K117/161/162 acetylation and K98 acetylation abrogates p53-mediated transcriptional regulation on SLC7A11, TIGAR, and GLS2 (32).

### Binding of p53 with DPP4 limits ferroptosis by regulating SLC7A11

Although p53 induces ferroptosis in a transcription-dependent manner in various cancers, in human colorectal cancer (CRC), it unusually functions in the regulation of erastin-mediated ferroptosis. p53-deficiency contributes to the increased lipid oxidation and GSH downregulation in CRC cells treated with erastin (42). Interestingly, the aforementioned alterations in malondialdehyde and GSH were recovered after transfecting p53 cDNA into p53^−/−^ CRC cells (42).

Depletion of p53 contributing to ferroptosis is involved with interdicting dipeptidyl-peptidase-4 (DPP4) activity in a transcription-independent mechanism. DPP4, a membrane-bound dimeric peptidase, is widely expressed in different cell types and can cleave and degrade various bioactive peptides biologically (43, 44). The function of DPP4 in tumorigenicity has been studied in many tumors (45). Deviant expression of DPP4 is associated with tumor aggressiveness in different cancers (18, 46). Paradoxically, some advanced malignancies, including lung squamous cell carcinoma and endometrial carcinoma, show the absence of DPP4 (22). Thus, DPP4 may play different roles in different backgrounds or cancers, and further studies are needed to elucidate the exact mechanism of DPP4 in cancer.

**Figure 3:**
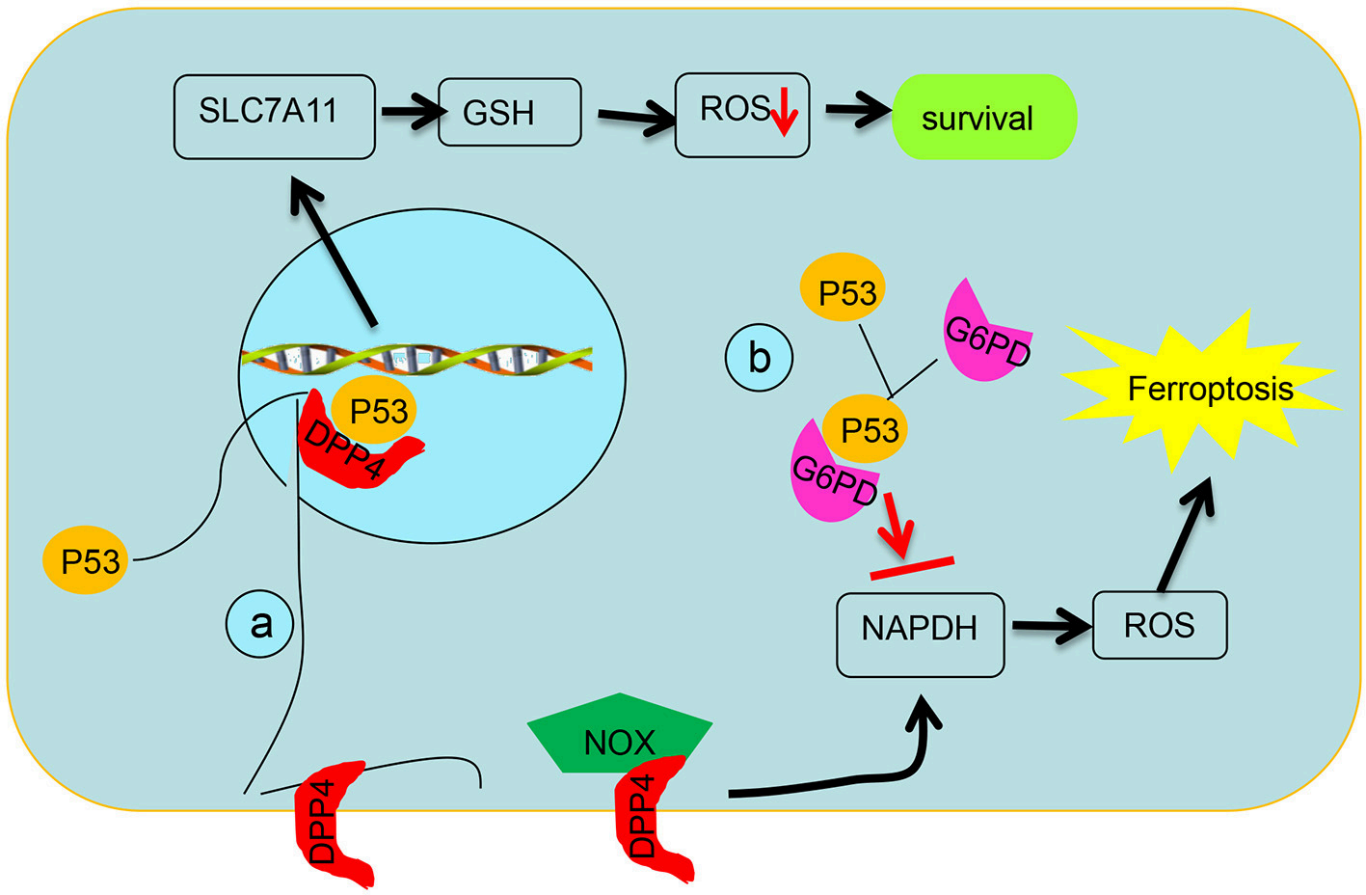
Schematic diagram of post-translational regulation of p53 on targets. **(a)** p53 antagonizes ferroptosis by favoring DPP4 into a nuclear to form of the DPP4–p53 complex and impeding formation of the DPP4–NOX complex, which is required for lipid peroxidation in ferroptosis. **(b)** p53 suppresses glucose metabolism and production of NADPH via inhibiting glucose-6-phosphate dehydrogenase directly.

DPP4 has been related to increased proportion of cancer stem cells and worse prognosis of CRC patients (16). Loss of p53 restrains the nuclear localization of DPP4 and boosts plasma-membrane-associated DPP4-dependent lipid peroxidation in CRC cells; then, the DPP4–NOX complex is formed and facilitates lipid peroxidation-induced ferroptosis. p53 antagonizes ferroptosis in CRC cells by facilitating DPP4 into nuclear to form the DPP4–p53 complex; dismantling of the DPP4–p53 complex can recover the ferroptosis sensitivity of CRC cells to erastin (Figure 3). This mechanism differs from the previously recognized role of p53 as a positive regulator of ferroptosis in non-CRC cells (13, 32, 47, 48). Thus, the bidirectional regulation of ferroptosis by p53 in a transcription-dependent and transcription-independent manner is dependent on tumor types and background.

However, many vital questions need to be elucidated. First, only two types of CRC cell lines are used in Xie's experiment (42), which is insufficient to prove the role of p53 and DPP4 on ferroptosis in CRC. Second, DPP4 is ubiquitously expressed in various cell types, including different tumors, whereas mutations and deletions of p53 are also common in malignant tumors. Further studies are needed to reveal the mechanism underlying the different roles of the DPP4–p53 complex on the regulation of SLC7A11 in CRC and other types of malignant tumors. Third, whether that p53 favors the localization of DPP4 into nuclear to form the DPP4–p53 complex could be affected by the mutation of p53 or modification of p53, such as acetylation, should be illuminated, and this may provide an answer to the opposite effects of p53 in different cellular context.

## P53 represses the TIGAR, GLS2, SCO2, and SAT1 genes to mediate ferroptosis

### Tigar plays an antioxidant functions in a p53-dependent manner

As a target of p53, TIGAR is prefigured to participate in tumor suppression and plays a role in antioxidant functions, which is in line with its functions in preventing cells from the acquirement of injury (49) (Figure 2). Nevertheless, in mouse models, the absence of TIGAR reduces capabilities to regenerate injured intestinal epithelium and represses tumor development with ROS restriction (50). TIGAR is upregulated in some cancer models and tumor types via a pattern that may be independent on the maintenance of p53 (51, 52). Furthermore, TIGAR expression negatively correlates with p53 expression in human breast cancer (53). p53-independent expression of TIGAR is poorly understood, although some transcription factors, such as SP1, CREB, and other members of the p53 family (p63 and p73), have been implicated in the regulation of p53 (17, 19, 54). In brief, these results highlight that TIGAR functions as a tumor suppressor in response to p53 but might also participate in cancer development when TIGAR expression is deregulated and uncoupled from p53 (20).

### GLS2 plays an antioxidant functions in a p53-dependent manner

Glutaminolysis plays crucial roles in ferroptosis (27). Glutaminolysis refers to the switch of glutamine into glutamate under the catalysis of GLS1 and GLS2. Although both enzymes are similar in structure and enzyme catalysis, GLS2 is required for ferroptosis (27). Human GLS2 gene is located on chromosome 12q13 and contains two potential p53 binding sites (BS). Adenovirus-mediated expression of p53 binds to both BS1 and BS2, but only BS2 is associated with endogenous p53. These data show that p53, once activated, can directly combine with BS2 in the GLS2 promoter and augment the mRNA expression of GLS2 (21). Upregulation of GLS2 contributes to p53-dependent ferroptosis by favoring aerobic glycolysis over oxidative phosphorylation and contributing to Warburg effect (27, 47, 55, 56) (Figure 2).

### p53-mediate metabolisms via repressing the SCO2

Synthesis of SCO2 is essential for regulating the cytochrome c oxidase complex, which is the main site of oxygen utilization in eukaryotic cells. The balance between the utilization of respiratory and glycolytic pathways is modulated by SCO2, which is a downstream target of p53 (57) (Figure 2). The source of energy from cellular respiration to glycolysis caused by the loss of p53 function resembles metabolic switch toward glycolysis in cancer cells with wild-type p53 when the SCO2 gene is depleted. SCO2 coupling p53 to mitochondrial respiration provides a possible interpretation for the Warburg phenomenon and supplies new ideas as to how p53 influences metabolism and ferroptosis (28).

## P53-mediated activation of SAT1 engages in ferroptosis

The polyamines, amino acid-derived polycationic alkylamines, are basic for the growth and survival of eukaryotic cells (58). Polyamine metabolism is frequently dysregulated in cancers (59). Spermidine/spermine N1-acetyltransferase 1 (SAT1), a rate-limiting enzyme, catalyzes the acetylation of spermidine and spermine into N1-acetylspermidine and N1-acetylspermine (60).

SAT1 could be highly induced by p53 (48). It is a p53-regulated target in wild-type p53 melanoma cells treated with Nutlin using RNA sequencing and two p53-binding sites have been found on the promoter region of SAT1. SAT1 transcriptionally activated in a p53-dependented manner is critical for lipid peroxidation and ROS-induced ferroptosis, and decreased expression of SAT1 significantly abrogates p53-induced ferroptosis. Elevation of prostaglandin-endoperoxide synthase 2 (PTGS2), a ferroptosis inducer, was identified in high-SAT1-expression xenograft tumors. Ferroptosis induced by SAT1 is arachidonate 15-lipoxygenase (ALOX15) dependent (Figure 2). ALOX15 is a lipoxygenase that catalyzes the peroxidation of arachidonic acid, and inhibition of ALOX15 can entirely rescue SAT1-induced ferroptosis. These results are consistent with the previous finding that ALOX15 is a main adjuster with which oxidative stress is transformed into lipid peroxidation and cell death (61). Nevertheless, whether that SAT1 plays a role in tumor suppression remains largely unknown.

## SOCS1 regulates ferroptosis by activating p53 via phosphorylation and stabilization

Suppressor of cytokine signaling (SOCS) family proteins have been implicated as negative feedback regulators of cytokine signaling pathways mediated by JAK-STAT (62). SOCS is involved in tumor development by regulating STATs in the background of aberrant activation of the JAK/STAT5 pathway. In particular, SOCS1 is thought to act as a pivotal tumor suppressor through negative regulation of JAKs and plays vital roles in tumor progression. Downregulated SOCS1 expression in various human cancers has been associated with dysregulation of cytokine receptor signaling pathways (63), whereas upregulated SOCS1 expression is associated with earlier tumor stages and better clinical outcomes in breast cancer (64).

A significant correlation exists between the expression of SOCS1 and the SOCS1-dependent p53 target genes in human fibroblasts, and SOCS1 is required for p53 activation (26, 65). SOCS1-regulated genes overlap with a set of genes induced by oxidized phospholipids, which has been recently linked to ferroptosis (66). The regulation of SAT1 by p53 is SOCS1-dependent, suggesting a role for SOCS1 in ferroptosis. Aside from influencing p53 target gene expression, SOCS1 also plays a general role in senescence by stabilizing the interactions of p53 with protein complexes at DNA damage foci (Figure 2). This function of SOCS1 allows the maintenance of a pool of preactive p53 that could be slowly released and contribute to generate a lasting chronic p53 response (67). SOCS1 activates the functions of p53 via facilitating the serine 15 phosphorylation of p53 and stabilizing p53 by interfering with KAP1 (67).

## Delayed ferroptosis onset requires p21

CDKN1A (encoding p21) is a well-characterized target of p53 and key mediator of p53-dependent cell-cycle progression. p21 upregulation could cause a coordinated p53-mediated response that normally decreases cystine import to match the lower metabolic demands of growth-arrested cells. The impact of p21 on GSH metabolism renders it a reasonable target for inducing ferroptosis in the context of p53 (68). Stabilization of p53 and activation of the p53–p21 axis make many cancer cells insensitive to ferroptosis induced by $system\;X_{c}^{-}$ inhibition or direct cystine deprivation. p21-dependent suppression of CDKs may be required to preserve GSH by regulating CDK-regulated metabolic enzymes and inhibit ferroptosis by inducing a complete cell-cycle arrest (69). However, the mechanism through which the p53–p21 axis reduces cellular reliance on $system\;X_{c}^{-}$-mediated cystine import and ongoing *de novo* GSH synthesis is unclear (36). Thus, the p53–p21 axis may help cancer cell survive metabolic stress, such as cystine deprivation, by suppressing the onset of ferroptosis, indicating that the p53–p21 transcriptional axis negatively regulates ferroptosis in cancer cells.

## S47 polymorphism of p53 decreases ferroptosis

Aside from mutations that impair p53 activity, single-nucleotide polymorphisms of p53 also alter cancer risk and clinical outcome significantly by impairing p53 signaling. About 20 years earlier, a naturally occurring polymorphism in p53 was discovered in Africans and African Americans; this polymorphism transforms the proline residue adjacent to Ser46 to a serine in human p53 (70). In particular, the Pro47Ser polymorphism (S47) impairs p53 apoptotic and transcriptional functions through reducing phosphorylation on serine 46 (47, 55). The defect in p53 function is traced to a restriction in downstream gene regulation that reduces cell ferroptosis in response to stress (70).

Profound cell death is induced in wild-type MEFs cells treated with erastin. However, cell viability assays certified that S47 MEFs and heterozygote S47/wild-type MEFs are resistant to erastin, especially S47 MEFs (47). Interestingly, treatment with erastin remarkably upregulates GLS2 expression in wild-type cells but not S47 cells, and depletion of GLS2 in wild-type MEFs recapitulates the cell death defect that is exhibited in S47 cells treated with erastin (47). The defective capacity of S47 to transactivate GLS2 might annotate the ferroptosis defect and tumor-prone characteristics of S47 mice (55).

In brief, elucidating the relevancy between p53 and ferroptosis has shown the other features of p53 biology and provided insights into the tumor suppression roles of p53. Clarification of the mechanism provides further insights into exploiting feasible therapeutic means through inducing ferroptosis defined by the occurrence of ROS in p53-retaining tumors. Nevertheless, the roles of p53 in ferroptosis remain formally demonstrated in different contexts due to the appearance of opposite effects in various cancer cells. Moreover, p53 could protect cells from slight stress damage via eliminating ROS, but p53-mediated ferroptosis owing to serious stress in cancer cells relies on the accumulation of ROS. Nevertheless, the mechanism of p53-mediated ROS production underlying cellular response is poorly understood.

## Author contributions

WZ and CG took part in the writing of the article. DD and FW participated in the data arrangement and drawing. WL examined and verified the article.

### Conflict of interest statement

The authors declare that the research was conducted in the absence of any commercial or financial relationships that could be construed as a potential conflict of interest.

## References

[1] Dixon SJ Lemberg KM Lamprecht MR Skouta R Zaitsev EM Gleason CE. Ferroptosis: an iron-dependent form of nonapoptotic cell death. Cell (2012) 149:1060–72. 10.1016/j.cell.2012.03.042 22632970 PMC3367386

[2] Xie Y Hou W Song X Yu Y Huang J Sun X. Ferroptosis: process and function. Cell Death Differ. (2016) 23:369–79. 10.1038/cdd.2015.158 26794443 PMC5072448

[3] Yagoda N von Rechenberg M Zaganjor E Bauer AJ Yang WS Fridman DJ Wolpaw AJ. RAS-RAF-MEK-dependent oxidative cell death involving voltage-dependent anion channels. Nature (2007) 447:864–8. 10.1038/nature05859 17568748 PMC3047570

[4] Conrad M Angeli JP Vandenabeele P Stockwell BR. Regulated necrosis: disease relevance and therapeutic opportunities. Nat Rev Drug Discov. (2016) 15:348–66. 10.1038/nrd.2015.6 26775689 PMC6531857

[5] Yang WS SriRamaratnam R Welsch ME Shimada K Skouta R Viswanathan VS. Regulation of ferroptotic cancer cell death by GPX4. Cell (2014) 156:317–31. 10.1016/j.cell.2013.12.010 24439385 PMC4076414

[6] Woo JH Shimoni Y Yang WS Subramaniam P Iyer A Nicoletti P. Elucidating compound mechanism of action by network perturbation analysis. Cell (2015) 162:441–51. 10.1016/j.cell.2015.05.056 26186195 PMC4506491

[7] Jackson JG Lozano G. The mutant p53 mouse as a pre-clinical model. Oncogene (2013) 32:4325–30. 10.1038/onc.2012.610 23318424

[8] Wang SJ Gu W. To be, or not to be: functional dilemma of p53 metabolic regulation. Curr Opin Oncol. (2014) 26:78–85. 10.1097/CCO.0000000000000024 24240177 PMC3968813

[9] Junttila MR Evan GI. p53–a Jack of all trades but master of none. Nat Rev Cancer (2009) 9:821–9. 10.1038/nrc2728 19776747

[10] Brady CA Jiang D Mello SS Johnson TM Jarvis LA Kozak MM. Distinct p53 transcriptional programs dictate acute DNA-damage responses and tumor suppression. Cell (2011) 145:571–83. 10.1016/j.cell.2011.03.035 21565614 PMC3259909

[11] Jiang D Brady CA Johnson TM Lee EY Park EJ Scott MP. Full p53 transcriptional activation potential is dispensable for tumor suppression in diverse lineages. Proc Natl Acad Sci USA. (2011) 108:17123–8. 10.1073/pnas.1111245108 21969549 PMC3193184

[12] Li T Kon N Jiang L Tan M Ludwig T Zhao Y. Tumor suppression in the absence of p53-mediated cell-cycle arrest, apoptosis, and senescence. Cell (2012) 149:1269–83. 10.1016/j.cell.2012.04.026 22682249 PMC3688046

[13] Jiang L Kon N Li T Wang SJ Su T Hibshoosh H. Ferroptosis as a p53-mediated activity during tumour suppression. Nature (2015) 520:57–62. 10.1038/nature14344 25799988 PMC4455927

[14] Torii S Shintoku R Kubota C Yaegashi M Torii R Sasaki M. An essential role for functional lysosomes in ferroptosis of cancer cells. Biochem J. (2016) 473:769–77. 10.1042/BJ20150658 26759376

[15] Hu W Zhang C Wu R Sun Y Levine A Feng Z. Glutaminase 2, a novel p53 target gene regulating energy metabolism and antioxidant function. Proc Natl Acad Sci USA. (2010) 107:7455–60. 10.1073/pnas.1001006107 20378837 PMC2867677

[16] Pang R Law WL Chu AC Poon JT Lam CS Chow AK. A subpopulation of CD26+ cancer stem cells with metastatic capacity in human colorectal cancer. Cell Stem Cell (2010) 6:603–15. 10.1016/j.stem.2010.04.001 20569697

[17] Zou S Gu Z Ni P Liu X Wang J Fan Q. SP1 plays a pivotal role for basal activity of TIGAR promoter in liver cancer cell lines. Mol Cell Biochem. (2012) 359:17–23. 10.1007/s11010-011-0993-0 21761199

[18] Yamaguchi U Nakayama R Honda K Ichikawa H Hasegawa T Shitashige M. Distinct gene expression-defined classes of gastrointestinal stromal tumor. J Clin Oncol. (2008) 26:4100–8. 10.1200/JCO.2007.14.2331 18757323

[19] Lee P Hock AK Vousden KH Cheung EC. p53- and p73-independent activation of TIGAR expression *in vivo*. Cell Death Dis. (2015) 6:e1842. 10.1038/cddis.2015.205 26247727 PMC4558498

[20] Gnanapradeepan K Basu S Barnoud T Budina-Kolomets A Kung CP Murphy ME. The p53 tumor suppressor in the control of metabolism and ferroptosis. Front Endocrinol. (2018) 9:124. 10.3389/fendo.2018.00124 29695998 PMC5904197

[21] Suzuki S Tanaka T Poyurovsky MV Nagano H Mayama T Ohkubo S. Phosphate-activated glutaminase (GLS2), a p53-inducible regulator of glutamine metabolism and reactive oxygen species. Proc Natl Acad Sci USA. (2010) 107:7461–6. 10.1073/pnas.1002459107 20351271 PMC2867754

[22] Kajiyama H Kikkawa F Ino K Shibata K Mizutani S. Expression of CD26/dipeptidyl peptidase IV in endometrial adenocarcinoma and its negative correlation with tumor grade. Adv Exp Med Biol. (2003) 524:245–8. 10.1007/0-306-47920-6_29 12675245

[23] Lo M Ling V Wang YZ Gout PW. The xc- cystine/glutamate antiporter: a mediator of pancreatic cancer growth with a role in drug resistance. Br J Cancer (2008) 99:464–72. 10.1038/sj.bjc.6604485 18648370 PMC2527809

[24] Schwartzenberg-Bar-Yoseph F Armoni M Karnieli E. The tumor suppressor p53 down-regulates glucose transporters GLUT1 and GLUT4 gene expression. Cancer Res. (2004) 64:2627–33. 10.1158/0008-5472.CAN-03-0846 15059920

[25] Kruse JP Gu W. Modes of p53 regulation. Cell (2009) 137:609–22. 10.1016/j.cell.2009.04.050 19450511 PMC3737742

[26] Mallette FA Calabrese V Ilangumaran S Ferbeyre G. SOCS1, a novel interaction partner of p53 controlling oncogene-induced senescence. Aging (2010) 2:445–52. 10.18632/aging.100163 20622265 PMC2933891

[27] Gao M Monian P Quadri N Ramasamy R Jiang X. Glutaminolysis and transferrin regulate ferroptosis. Mol Cell (2015) 59:298–308. 10.1016/j.molcel.2015.06.011 26166707 PMC4506736

[28] Matoba S Kang JG Patino WD Wragg A Boehm M Gavrilova O. p53 regulates mitochondrial respiration. Science (2006) 312:1650–3. 10.1126/science.1126863 16728594

[29] Bensaad K Tsuruta A Selak MA Vidal MN Nakano K Bartrons R. TIGAR, a p53-inducible regulator of glycolysis and apoptosis. Cell (2006) 126:107–20. 10.1016/j.cell.2006.05.036 16839880

[30] Jiang P Du W Wang X Mancuso A Gao X Wu M. p53 regulates biosynthesis through direct inactivation of glucose-6-phosphate dehydrogenase. Nat Cell Biol. (2011) 13:310–6. 10.1038/ncb2172 21336310 PMC3110666

[31] Conrad M Sato H. The oxidative stress-inducible cystine/glutamate antiporter, system x (c) (-) : cystine supplier and beyond. Amino Acids (2012) 42:231–46. 10.1007/s00726-011-0867-5 21409388

[32] Wang SJ Li D Ou Y Jiang L Chen Y Zhao Y. Acetylation is crucial for p53-mediated ferroptosis and tumor suppression. Cell Rep. (2016) 17:366–73. 10.1016/j.celrep.2016.09.022 27705786 PMC5227654

[33] Bridges RJ Natale NR Patel SA. System xc(-) cystine/glutamate antiporter: an update on molecular pharmacology and roles within the CNS. Br J Pharmacol. (2012) 165:20–34. 10.1111/j.1476-5381.2011.01480.x 21564084 PMC3252963

[34] Dixon SJ Patel DN Welsch M Skouta R Lee ED Hayano M. Pharmacological inhibition of cystine-glutamate exchange induces endoplasmic reticulum stress and ferroptosis. Elife (2014) 3:e02523. 10.7554/eLife.02523 24844246 PMC4054777

[35] Gupta AK Bharadwaj M Kumar A Mehrotra R. Spiro-oxindoles as a promising class of small molecule inhibitors of p53-MDM2 interaction useful in targeted cancer therapy. Top Curr Chem. (2017) 375:3. 10.1007/s41061-016-0089-0 27943171

[36] Tarangelo A Magtanong L Bieging-Rolett KT Li Y Ye J Attardi LD. p53 suppresses metabolic stress-induced ferroptosis in cancer cells. Cell Rep. (2018) 22:569–75. 10.1016/j.celrep.2017.12.077 29346757 PMC5791910

[37] Galluzzi L Bravo-San Pedro JM Kroemer G. Ferroptosis in p53-dependent oncosuppression and organismal homeostasis. Cell Death Differ. (2015) 22:1237–8. 10.1038/cdd.2015.54 26143748 PMC4495364

[38] Ying Y Padanilam BJ. Regulation of necrotic cell death: p53, PARP1 and cyclophilin D-overlapping pathways of regulated necrosis? Cell Mol Life Sci. (2016) 73:2309–24. 10.1007/s00018-016-2202-5 27048819 PMC5490387

[39] Eischen CM Lozano G. The Mdm network and its regulation of p53 activities: a rheostat of cancer risk. Hum Mutat. (2014) 35:728–37. 10.1002/humu.22524 24488925 PMC4725600

[40] Sykes SM Mellert HS Holbert MA Li K Marmorstein R Lane WS. Acetylation of the p53 DNA-binding domain regulates apoptosis induction. Mol Cell (2006) 24:841–51. 10.1016/j.molcel.2006.11.026 17189187 PMC1766330

[41] Tang Y Zhao W Chen Y Zhao Y Gu W. Acetylation is indispensable for p53 activation. Cell (2008) 133:612–26. 10.1016/j.cell.2008.03.025 18485870 PMC2914560

[42] Xie Y Zhu S Song X Sun X Fan Y Liu J. The tumor suppressor p53 limits ferroptosis by blocking DPP4 activity. Cell Rep. (2017) 20:1692–704. 10.1016/j.celrep.2017.07.055 28813679

[43] Liang PI Yeh BW Li WM Chan TC Chang IW Huang CN. DPP4/CD26 overexpression in urothelial carcinoma confers an independent prognostic impact and correlates with intrinsic biological aggressiveness. Oncotarget (2017) 8:2995–3008. 10.18632/oncotarget.13820 27936466 PMC5356858

[44] Carl-McGrath S Lendeckel U Ebert M Rocken C. Ectopeptidases in tumour biology: a review. Histol Histopathol. (2006) 21:1339–53. 10.14670/HH-21.1339 16977585

[45] Cordero OJ Salgado FJ Nogueira M. On the origin of serum CD26 and its altered concentration in cancer patients. Cancer Immunol Immunother. (2009) 58:1723–47. 10.1007/s00262-009-0728-1 19557413 PMC11031058

[46] Stremenova J Krepela E Mares V Trim J Dbaly V Marek J. Expression and enzymatic activity of dipeptidyl peptidase-IV in human astrocytic tumours are associated with tumour grade. Int J Oncol. (2007) 31:785–92. 10.3892/ijo.31.4.785 17786309

[47] Jennis M Kung CP Basu S Budina-Kolomets A Leu JI Khaku S. An African-specific polymorphism in the TP53 gene impairs p53 tumor suppressor function in a mouse model. Genes Dev. (2016) 30:918–30. 10.1101/gad.275891.115 27034505 PMC4840298

[48] Ou Y Wang SJ Li D Chu B Gu W. Activation of SAT1 engages polyamine metabolism with p53-mediated ferroptotic responses. Proc Natl Acad Sci USA. (2016) 113:E6806–12. 10.1073/pnas.1607152113 27698118 PMC5098629

[49] Rajendran R Garva R Ashour H Leung T Stratford I Krstic-Demonacos M. Acetylation mediated by the p300/CBP-associated factor determines cellular energy metabolic pathways in cancer. Int J Oncol. (2013) 42:1961–72. 10.3892/ijo.2013.1907 23591450

[50] Cheung EC Athineos D Lee P Ridgway RA Lambie W Nixon C. TIGAR is required for efficient intestinal regeneration and tumorigenesis. Dev Cell (2013) 25:463–77. 10.1016/j.devcel.2013.05.001 23726973 PMC3682186

[51] Li B Wang Z Xie JM Wang G Qian LQ Guan XM. TIGAR knockdown enhanced the anticancer effect of aescin via regulating autophagy and apoptosis in colorectal cancer cells. Acta Pharmacol Sin. (2018). [Epub ahead of print]. 10.1038/s41401-018-0001-2 29769743 PMC6318299

[52] Shen M Zhao X Zhao L Shi L An S Huang G. Met is involved in TIGAR-regulated metastasis of non-small-cell lung cancer. Mol Cancer (2018) 17:88. 10.1186/s12943-018-0839-4 29753331 PMC5948872

[53] Won KY Lim SJ Kim GY Kim YW Han SA Song JY. Regulatory role of p53 in cancer metabolism via SCO2 and TIGAR in human breast cancer. Hum Pathol. (2012) 43:221–8. 10.1016/j.humpath.2011.04.021 21820150

[54] Zou S Wang X Deng L Wang Y Huang B Zhang N. CREB, another culprit for TIGAR promoter activity and expression. Biochem Biophys Res Commun. (2013) 439:481–6. 10.1016/j.bbrc.2013.08.098 24036271

[55] Basu S Barnoud T Kung CP Reiss M Murphy ME. The African-specific S47 polymorphism of p53 alters chemosensitivity. Cell Cycle (2016) 15:2557–60. 10.1080/15384101.2016.1215390 27484708 PMC5053554

[56] Zhang C Liu J Liang Y Wu R Zhao Y Hong X. Tumour-associated mutant p53 drives the Warburg effect. Nat Commun. (2013) 4:2935. 10.1038/ncomms3935 24343302 PMC3969270

[57] Qi Z He J Su Y He Q Liu J Yu L. Physical exercise regulates p53 activity targeting SCO2 and increases mitochondrial COX biogenesis in cardiac muscle with age. PLoS ONE (2011) 6:e21140. 10.1371/journal.pone.0021140 21750704 PMC3131270

[58] Gerner EW Meyskens FL Jr. Polyamines and cancer: old molecules, new understanding. Nat Rev Cancer (2004) 4:781–92. 10.1038/nrc1454 15510159

[59] Casero RA Jr Marton LJ. Targeting polyamine metabolism and function in cancer and other hyperproliferative diseases. Nat Rev Drug Discov. (2007) 6:373–90. 10.1038/nrd2243 17464296

[60] Pegg AE. Spermidine/spermine-N(1)-acetyltransferase: a key metabolic regulator. Am J Physiol Endocrinol Metab. (2008) 294:E995–1010. 10.1152/ajpendo.90217.2008 18349109

[61] Shintoku R Takigawa Y Yamada K Kubota C Yoshimoto Y Takeuchi T. Lipoxygenase-mediated generation of lipid peroxides enhances ferroptosis induced by erastin and RSL3. Cancer Sci. (2017) 108:2187–94. 10.1111/cas.13380 28837253 PMC5666033

[62] Slattery ML Lundgreen A Kadlubar SA Bondurant KL Wolff RK. JAK/STAT/SOCS-signaling pathway and colon and rectal cancer. Mol Carcinogen. (2013) 52:155–66. 10.1002/mc.21841 22121102 PMC3430812

[63] Jiang M Zhang WW Liu P Yu W Liu T Yu J. Dysregulation of SOCS-mediated negative feedback of cytokine signaling in carcinogenesis and its significance in cancer treatment. Front Immunol. (2017) 8:70. 10.3389/fimmu.2017.00070 28228755 PMC5296614

[64] Sasi W Jiang WG Sharma A Mokbel K. Higher expression levels of SOCS 1,3,4,7 are associated with earlier tumour stage and better clinical outcome in human breast cancer. BMC Cancer (2010) 10:178. 10.1186/1471-2407-10-178 20433750 PMC2876081

[65] Calabrese V Mallette FA Deschenes-Simard X Ramanathan S Gagnon J Moores A. SOCS1 links cytokine signaling to p53 and senescence. Mol Cell (2009) 36:754–67. 10.1016/j.molcel.2009.09.044 20005840

[66] Kagan VE Mao G Qu F Angeli JP Doll S Croix CS. Oxidized arachidonic and adrenic PEs navigate cells to ferroptosis. Nat Chem Biol. (2017) 13:81–90. 10.1038/nchembio.2238 27842066 PMC5506843

[67] Saint-Germain E Mignacca L Vernier M Bobbala D Ilangumaran S Ferbeyre G. SOCS1 regulates senescence and ferroptosis by modulating the expression of p53 target genes. Aging (2017) 9:2137–62. 10.18632/aging.101306 29081404 PMC5680560

[68] Maddocks OD Berkers CR Mason SM Zheng L Blyth K Gottlieb E. Serine starvation induces stress and p53-dependent metabolic remodelling in cancer cells. Nature (2013) 493:542–6. 10.1038/nature11743 23242140 PMC6485472

[69] Ewald JC Kuehne A Zamboni N Skotheim JM. The yeast cyclin-dependent kinase routes carbon fluxes to fuel cell cycle progression. Mol Cell (2016) 62:532–45. 10.1016/j.molcel.2016.02.017 27203178 PMC4875507

[70] Lane D. p53: out of Africa. Genes Dev. (2016) 30:876–77. 10.1101/gad.281733.116 27083994 PMC4840293

